# Optimization of Aluminum Stressed Skin Panels in Offshore Applications

**DOI:** 10.3390/ma7096811

**Published:** 2014-09-19

**Authors:** Dianne van Hove, Frans Soetens

**Affiliations:** Department of the Built Environment, Unit Structural Design, Eindhoven University of Technology, Postbox 513, 5600 MB Eindhoven, The Netherlands; E-Mail: f.soetens@tue.nl

**Keywords:** stressed skin panels, hat stiffeners, aluminum offshore living quarters

## Abstract

Since the introduction of general European rules for the design of aluminium structures, specific rules for the design of aluminum stressed skin panels are available. These design rules have been used for the optimization of two extrusion products: one for explosions and wind load governing and one for explosions and floor load governing. The optimized extrusions fulfill Class 3 section properties, leading to weight reductions up to 25% of regularly-used shear panel sections. When the design is based on Class 4 section properties, even more weight reduction may be reached. The typical failure mode of the optimized stressed skin panels depends on the applied height of the hat stiffeners. For sections using relatively high hat stiffeners, failure is introduced by yielding of the heat-affected zone. For this type of cross-section, Eurocode 9 design rules and numerical calculations show very good agreement. For sections using relatively low hat stiffeners, failure is introduced by global buckling. For this type of cross-section, Eurocode 9 gives rather conservative results.

## 1. Introduction

For many years, steel, as well as aluminum alloys have been used as a load bearing material in the structural design of helicopter decks, platforms, bridges and ships. Nowadays, also living quarters on oil platforms make use of aluminum as the main structural material. The main reasons for this application are its low weight, as well as its excellent corrosion resistance during its lifetime in unfavorable environmental conditions.

Until now, aluminum structures in living quarters on platforms have been designed using national guidelines, mainly based on experience, as well as design rules for steel structures. However, since the introduction of Eurocode 9 [1], specific rules have been available for the design of aluminum stressed skin panels. These shear panels are often used for the stabilization of frames, as used in living quarters on platforms.

In this research, the design of aluminum stressed skin panels is optimized using the design regulations in Eurocode 9 [1].

## 2. Design Conditions

The design conditions for the investigated stressed skin panels were categorized into structural conditions and cross-sectional (extrusion product) conditions.

Considering the structural conditions, aluminum alloy AA6082-T6 was chosen for its beneficial properties: good corrosion resistance, favorable mechanical properties, good behavior of connections under dynamic loading conditions and ability for friction stir welding. The last aspect was considered as an important criterion, because of the beneficial properties of the friction stir welding (FSW) connection method. If the cross-section is designed well for FSW, the welding speed is relatively great, which significantly reduces the connection costs.

**Figure 1:**
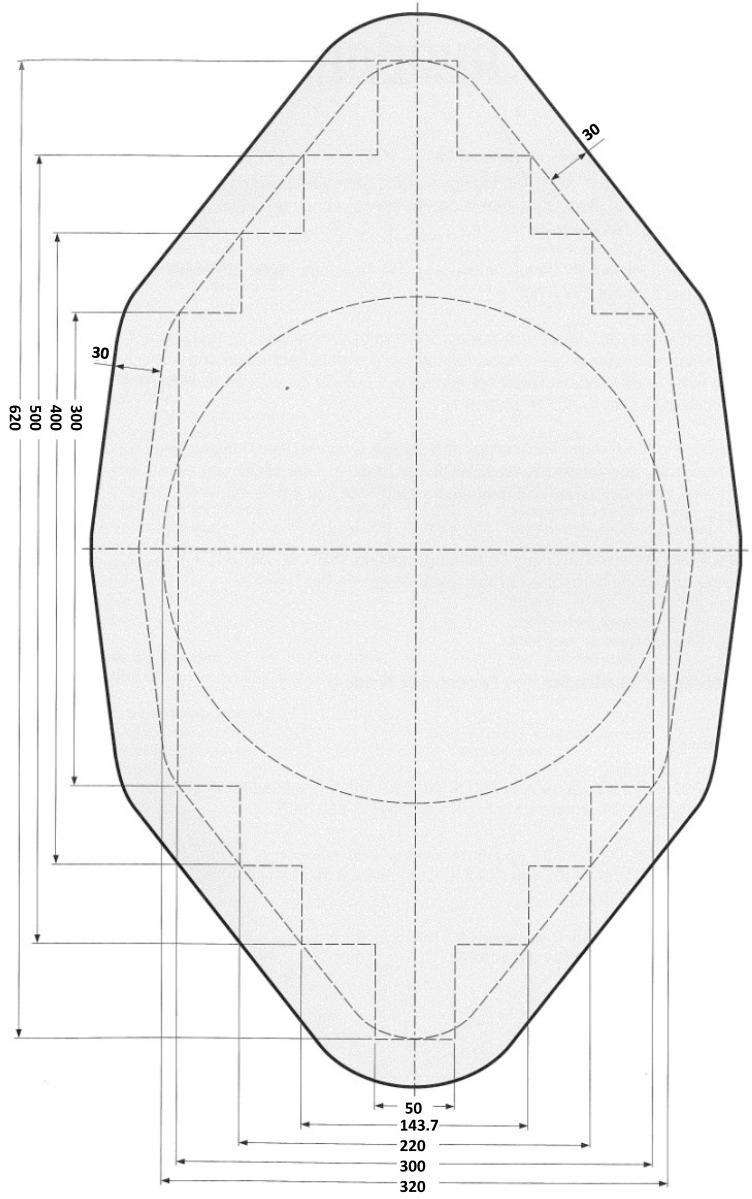
Die geometry (measures in mm).

Further, considering the cross-sectional conditions, the panels were supposed to be composed of aluminum extrusions, which can be produced by a die fulfilling the geometrical conditions given in Figure 1. The maximum width of the cross-section is 620 mm. Further, several cross-sectional conditions concerning the weldability should be met, such as:

- the connections are realized by double-sided welding, which improves the quality of the connection according to construction practice;
- the weld angles in the extrusion product are smaller than 90 degrees, which facilitates good grinding out of the weld geometry;
- the number of welds is minimized, which implies a large width of the section to be extruded;
- the plates to be connected have equal thicknesses, which best facilitates the friction stir welding method to be used;
- the minimum wall thickness of the cross-sectional parts is 2.5 mm, which guarantees extrusion products having small dimensional deviations (tolerances).

**Figure 2:**
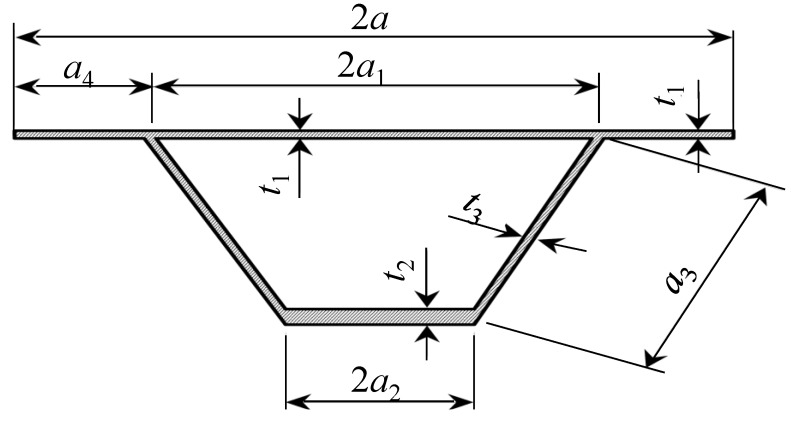
Basic cross-sectional dimensions of a hat profiled shear panel section where: *a*, overall top flange width; *a*_1_, internal top flange width; *a*_2_, internal bottom flange width; *a*_3_, internal web height; *a*_4_, external top flange width; *t*_1_, top flange thickness; *t*_2_, bottom flange thickness; *t*_3_, web thickness.

From extended literature studies [2,3], it is concluded that hat profiles, as shown in Figure 2, are most efficient when comparing minimum weight *versus* maximum strength. Only these types of cross-sections where investigated further.

**Figure 3:**
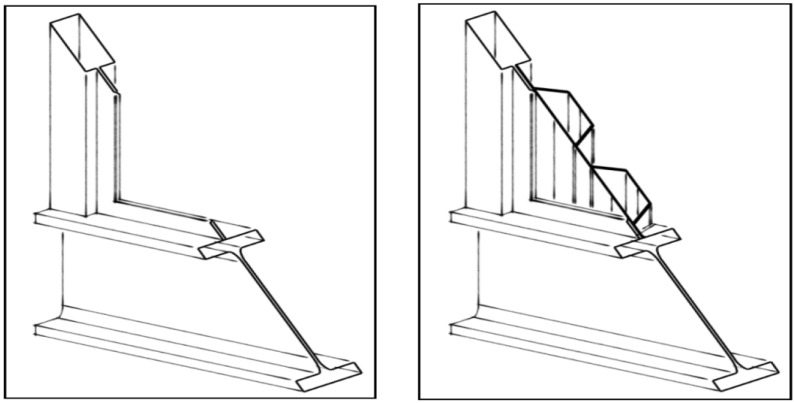
Frames without shear panels and frames with shear panels, respectively.

The extrusions are welded together to arrive at a shear panel using friction stir welding (see Figure 3). This welding procedure enables high speeds, which reduce the costs of the welds. For the strength properties of the friction stir welds, the design strengths proposed by Ogle [4] are used (Table 1), where: f_o;d_, design value of the 0.2% strain strength; f_u;d_, design value of the ultimate tensile strength; ε_u_, ultimate strain limit.

**Table 1:** Material properties of alloy AA6082-T6 and friction stir welded (FSW) welds.

Material	f_o;d_ (N/mm^2^)	f_u;d_ (N/mm^2^)	ε_u_ in %
Extrusions: AA6082-T6	250	290	8.00
Welds: FSW	160	254	4.85

Panel measurements are derived from a standard housing depth, including services of 4 m; the width of the panels is 4 m, as well. The panels are welded on both sides of the main bearing structures, usually built up by I-sections, using the metal inert gas (MIG) welding procedure. These welded connections can be schematized as hinges (see Figure 2).

The panels are designed for load configurations parallel to the plane, as well as load combinations perpendicular to the plane. The loads can be divided into the following categories: weight, wind loading, floor loading and explosions. The loads, safety factor and load combinations are according to [5].

## 3. Optimization Procedure

Using the design conditions mentioned in Section 2 and using hat profiles as the most efficient cross-section (see [3]), the optimization for extrusion measurements (width, height and thickness) has been carried out for different loading conditions. For the determination of the bending moment capacity, as well as the in-plane shear capacity, strength calculations have been carried out according to [1]. Furthermore, stiffness calculations have been carried out for the determination of the required bending stiffness (see [2] for further details).

The optimization procedure for the determination of the bending moment capacity, as well as the in-plane shear capacity is as follows:

- global determination of the required thickness of the upper flange to be able to start the optimization procedure;
- set minimum wall thickness of all cross-section parts equal to 2.5 mm;
- determine cross-sectional limits to fulfill Class 3 conditions according to [1];
- calculate a unity check for moment capacity or in-plane shear capacity according to [1];
- repeat procedure until the unity check equals one.

Regarding the profile for in-plane loading, the optimal in-plane shear capacity using the minimum required wall thickness is 2.5 mm, and two different iteration procedures are necessary. In one procedure, the reduced shear strength of the hat stiffeners (ρ_c;g_) is set equal to the reduced heat affected-zone strength due to welding (ρ_o,HAZ_). Additionally, in the other procedure, the wall thickness is minimized, which reduces the stiffness of the hat stiffener.

Regarding the profile for out-of-plane loading, the same kind of optimization procedure can be followed, which results in a minimal cross-section for each load configuration.

**Figure 4:**
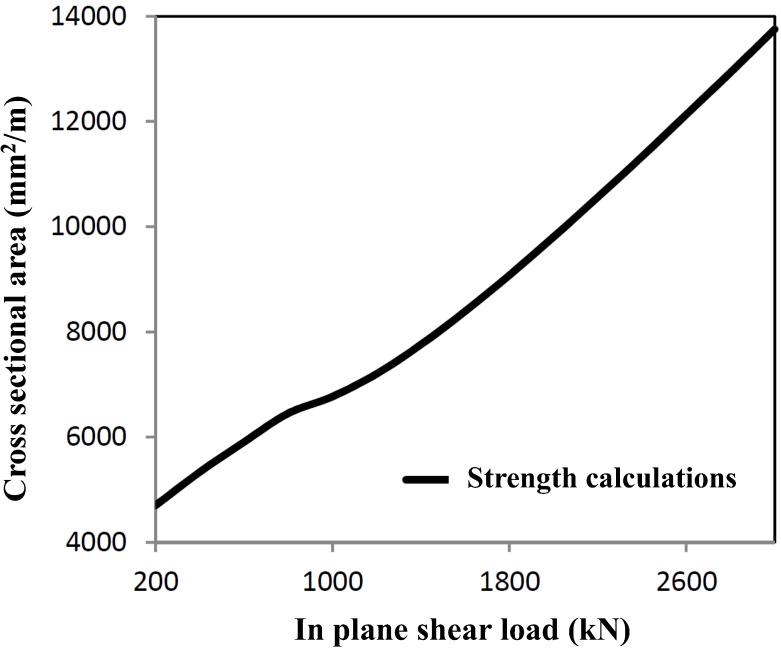
Minimum cross-section necessary for shear load combinations.

For in-plane stiffness and strength, the shear plane loads are decisive for optimal profile measurements. The calculations have been worked out for a shear panel of 4 × 4 m^2^, resulting in the minimum cross-sectional area, as given in Figure 4.

**Figure 5:**
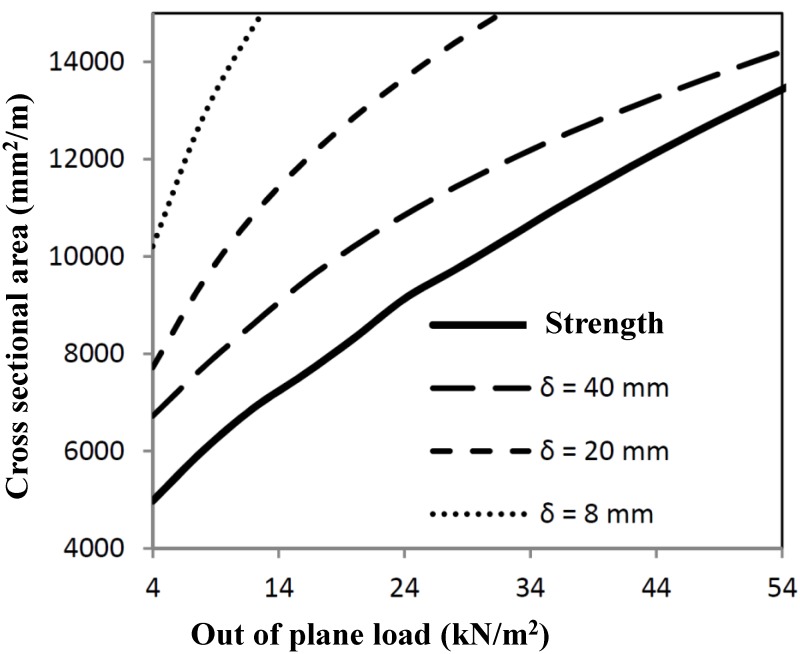
Minimum cross-section necessary for out-of-plane load combinations, where δ is the considered maximum allowable out-of-plane deformation.

The same has been done for the case that out-of-plane loading (for example explosions) is governing. These calculations have also been worked out for a panel of 4 × 4 m^2^, resulting in the minimum cross-sectional area, as given in Figure 5, fulfilling strength conditions, as well as deformation conditions.

**Figure 6:**
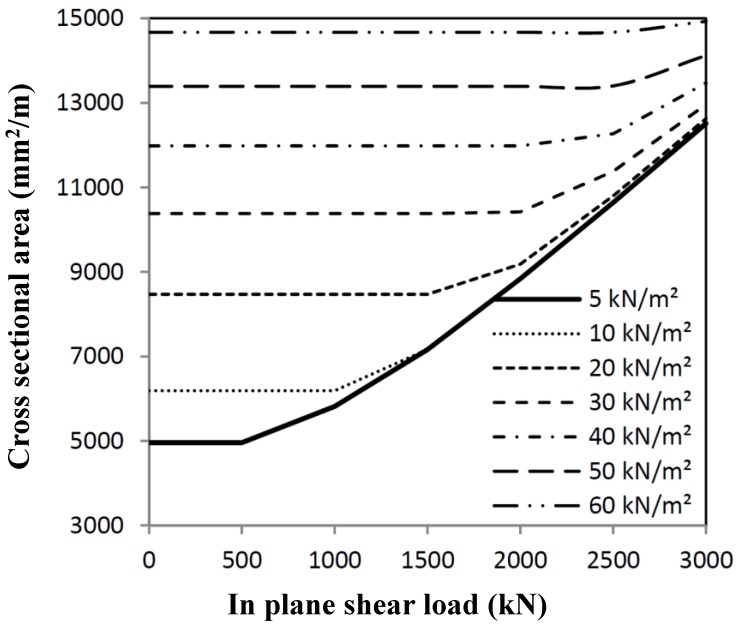
Minimum cross-sectional area for out-of-plane loading dependent on the shear load.

**Figure 7:**
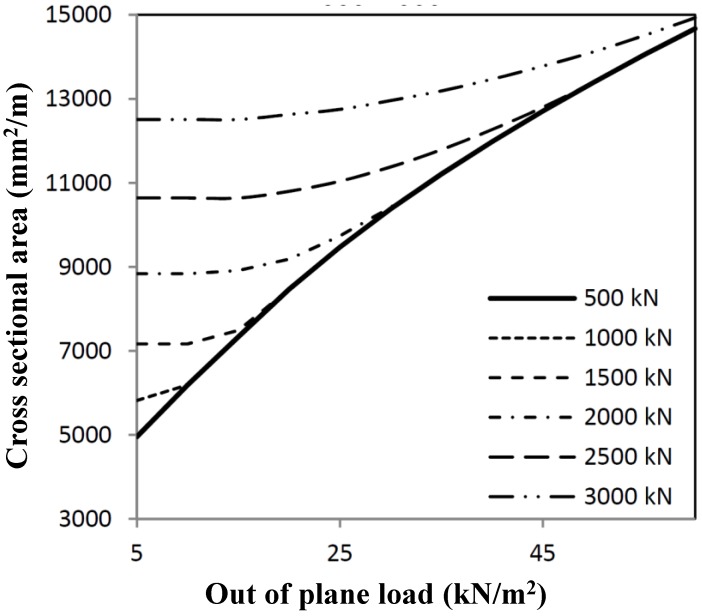
Minimum cross-sectional area for shear loading dependent on the out-of-plane load.

Loads have to be combined for several load combinations. The interaction of both optimization procedures results in interaction graphs, as shown in Figure 6 and Figure 7, in which strength calculations have been mixed. The optimum cross-section can be derived from the combination of the shear load and out-of-plane load. When deformations are relevant (see Figure 5), then the minimum area will be governed by out-of-plane loading, dependent on the deformation criterion used.

The design of the stressed skin panels is based on an application in a six-story living quarters with a height of 24 m (six panels) and a floor area of eight by 12 m (two by three panels). The design loads are according to [1,5] worked out for three different loadings:

- permanent loading 2.0 kN/m^2^ (weight, services and floor finishing);
- variable loading (wind 2.0 kN/m^2^ , floor 5.0 kN/m^2^);
- special loadings (explosions 10 kN/m^2^ or 25 kN/m^2^, based on [5]).

Four governing panels have been investigated:

- wall panel loaded by static pressure due to explosions 25 kN/m^2^;
- wall panel loaded by static pressure due to explosions 10 kN/m^2^;
- floor panel;
- combination panel.

The aluminum alloy used is AA-6082-T6, having a designed 0.2% yield strength f_0,d_ = 250 N/mm^2^, a heat affected zone (HAZ) strength f_0,HAZ,d_ = 160 N/mm^2^ or a HAZ factor ρ_HAZ_ = 0.64, according to Eurocode 9 [1]. The length of the HAZ zone equals 20 mm. Deformation limits are set to 20 mm (0.5% of the span length) for total deflections δ_max_ and 13.3 mm (0.33% of span length) for additional deflections δ_2_.

For load combinations, including explosions, the serviceability limit states are not taken into account. For all other load combinations, ultimate limit states, as well as serviceability limit states are relevant.

## 4. Optimization of Cross-Section

**Figure 8:**
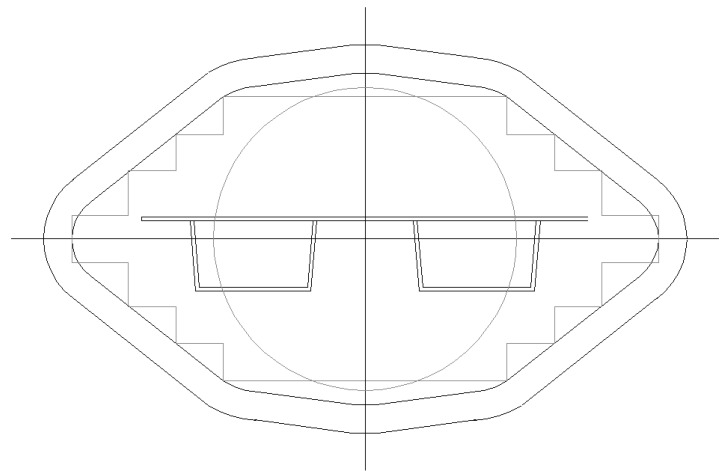
Optimized Panel Section 1 (see Figure 2 for an explanation).

**Figure 9:**
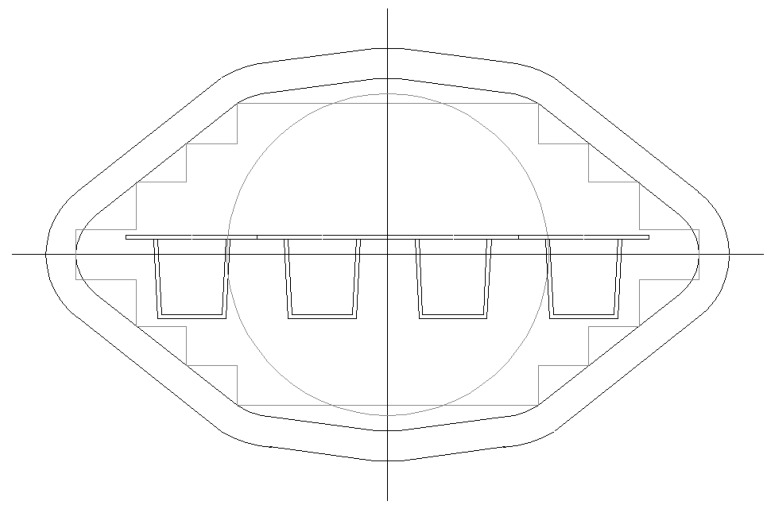
Optimized Panel Section 1 (see Figure 2 for an explanation).

**Figure 10:**
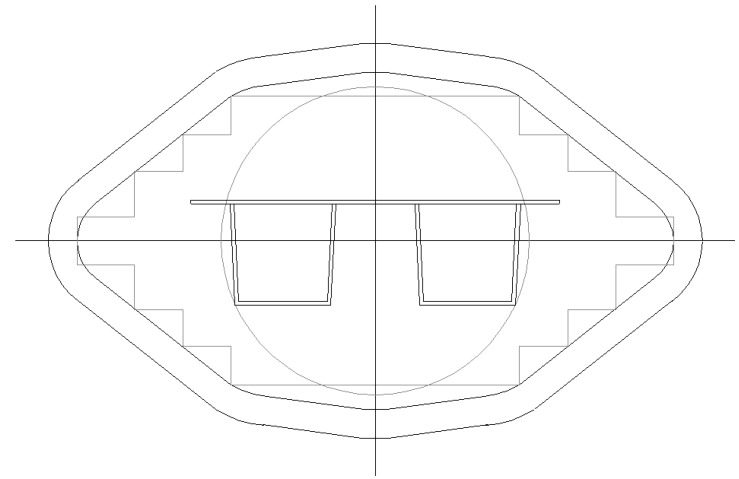
Optimized Panel Section 3 (see Figure 3).

**Figure 11:**
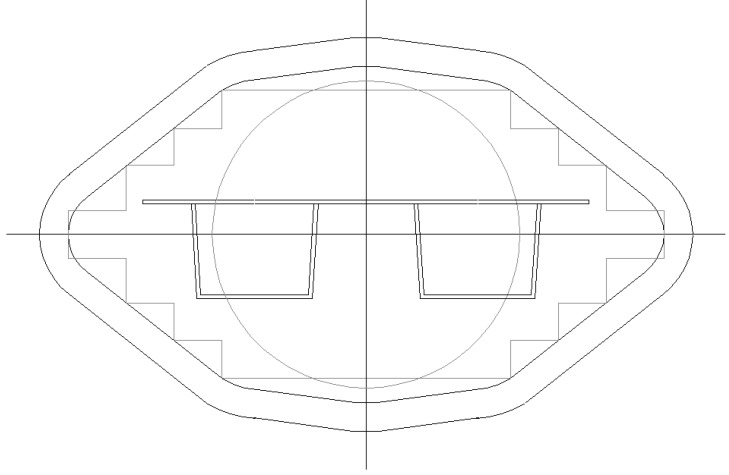
Optimized Panel Section 3 (see Figure 4).

Strength and stiffness calculations according to Eurocode 9 [1] have resulted in optimized panels fitting maximum extrusion dimensions. The following optimal cross-sections can be distinguished (Figure 8, Figure 9, Figure 10 and Figure 11):

- Panel 1 optimized for explosions 25 kN/m^2^ and wind loading 2.0 kN/m^2^, see Table 2, Table 3, Figure 8;
- Panel 2 optimized for explosions 10 kN/m^2^ and wind loading 2.0 kN/m^2^, see Table 4, Table 5, Figure 9;
- Panel 3 optimized for self-weight 2.0 kN/m^2^ and floor loading 5.0 kN/m^2^, see Table 6, Table 7, Figure 10;
- Panel 4 optimized for load conditions of Panel 1 (thickness of upper plate) and load conditions of Panel 3 (hat stiffener of Panel 3), see Table 8, Table 9, Figure 11.

**Table 2:** Dimensions of optimized Panel Section 1 (see Figure 2 for an explanation).

Panel Section 1	Length	Thickness
*a*	118 mm	–
*h*	74 mm	–
*a*_1_	65 mm	–
*a*_2_	59 mm	–
*a*_3_	74 mm	–
*a*_4_	53 mm	–
*t*_1_	–	4.9 mm
*t*_2_	–	5.3 mm
*t*_3_	–	2.5 mm

**Table 3:** Section properties of optimized Panel Section 1 (see Figure 2).

Panel section 1	Section property	Reduction factor
*A*	9,124 mm^2^/m	–
*W*_eff_	52 × 10^3^ mm^3^	–
*I*_eff_	231.5 × 10^4^ mm^4^	–
ρ**_c;g_**	–	0.81
ρ_o,HAZ_	–	0.64

**Table 4:** Dimensions of optimized Panel Section 1 (see Figure 2 for an explanation).

Panel Section 2	Length	Thickness
*a*	64 mm	–
*h*	79 mm	–
*a*_1_	35 mm	–
*a*_2_	32 mm	–
*a*_3_	79 mm	–
*a*_4_	29 mm	–
*t*_1_	–	2.7 mm
*t*_2_	–	2.9 mm
*t*_3_	–	2.5 mm

**Table 5:** Section properties of optimized Panel Section 1 (see Figure 2).

Panel Section 2	Section property	Reduction factor
*A*	7,240 mm^2^/m	–
*W*_eff_	21 × 10^3^ mm^3^	–
*I*_eff_	93 × 10^4^ mm^4^	–
ρ**_c;g_**	–	0.79
ρ_o,HAZ_	–	0.64

**Table 6:** Dimensions of optimized Panel Section 3 (see Figure 2 for an explanation).

Panel Section 3	Length	Thickness
*a*	96 mm	–
*h*	105 mm	–
*a*_1_	53 mm	–
*a*_2_	48 mm	–
*a*_3_	106 mm	–
*a*_4_	43 mm	–
*t*_1_	–	4.1 mm
*t*_2_	–	4.4 mm
*t*_3_	–	2.7 mm

**Table 7:** Section properties of optimized Panel Section 3 (see Figure 2).

Panel Section 3	Section property	Reduction factor
*A*	9,234 mm^2^/m	–
*W*_eff_	57 × 10^3^ mm^3^	–
*I*_eff_	350 × 10^4^ mm^4^	–
ρ**_c;g_**	–	1.00
ρ_o,HAZ_	–	0.64

**Table 8:** Dimensions of optimized Panel Section 4 (see Figure 2 for an explanation).

Panel Section 3	Length	Thickness
*a*	116 mm	–
*h*	99 mm	–
*a*_1_	64 mm	–
*a*_2_	58 mm	–
*a*_3_	99 mm	–
*a*_4_	52 mm	–
*t*_1_	–	4.9 mm
*t*_2_	–	5.3 mm
*t*_3_	–	2.5 mm

**Table 9:** Section properties of optimized Panel Section 4 (see Figure 2).

Panel Section 3	Section property	Reduction factor
*A*	9,742 mm^2^/m	–
*W*_eff_	72 × 10^3^ mm^3^	–
*I*_eff_	2427 × 10^4^ mm^4^	–
ρ**_c;g_**	–	1.00
ρ_o,HAZ_	–	0.64

A comparison of the optimized cross-sections of Panels 1 and 2 with existing shear panels [6] results in a 10% to 25% weight reduction. Most of the weight reduction is realized by the optimized dimensions of the hat stiffener. It should be mentioned that even more weight reduction could be realized by designing Class 4 cross-sections instead of Class 3 cross-sections. However, in that case, production limits, fabrication limits and erection limits for very slender section parts should be taken into account.

## 5. FEM Analysis

**Figure 12:**
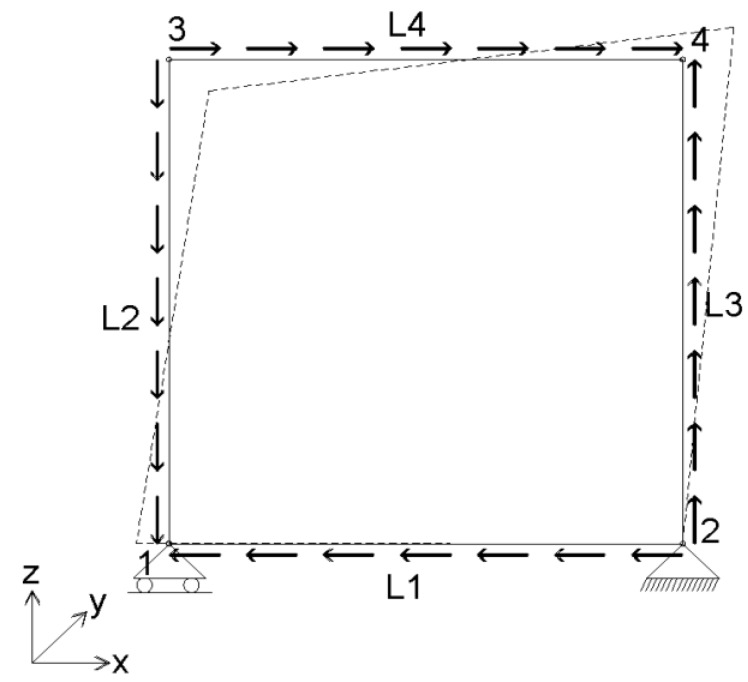
In-plane shear loading using line loads L.

**Figure 13:**
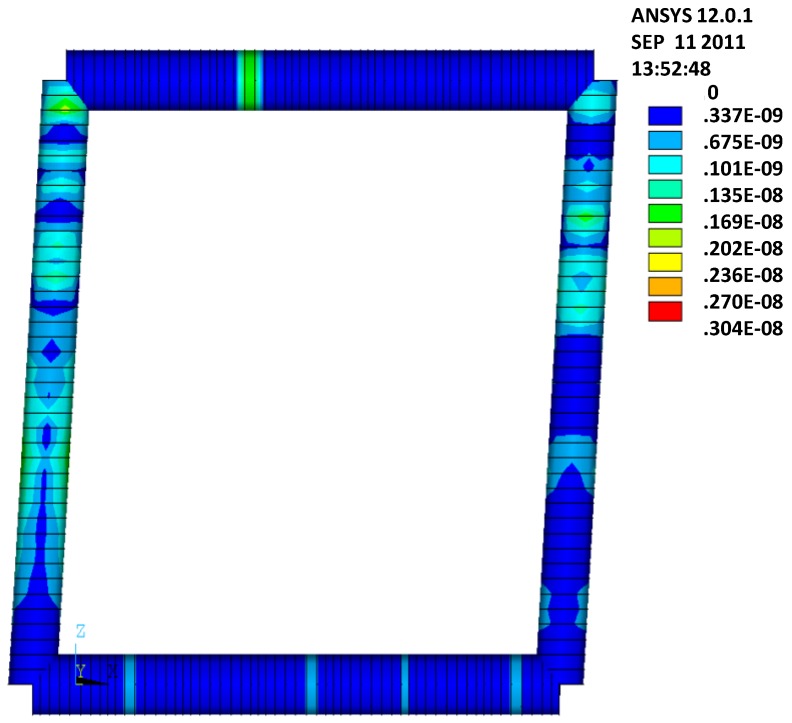
Stresses in a structure without shear panels and with arbitrary edge beams.

For the verification of the analytical results, an FEM analysis using ANSYS version 12.0.1 has been carried out. The infill hat profiled plates have been simulated using SHELL181 elements, and the edge beams of the frame have been simulated using BEAM188 elements. As the geometry of the edge beam is unknown, the BEAM elements are introduced by using arbitrary section types (ASEC), which facilitate introducing arbitrary geometric properties. Further, the ANSYS model is built in three layers, where the panel is modeled in the first layer and the horizontal and vertical edge beams are modeled in the second and third layer, respectively. To prevent concentrated stresses in the panel, the shear loads are introduced using line loading (see Figure 12). Further, to check the boundary conditions supplied by the supports and the edge beams (hinges in the corners), a separate model without plates is analyzed for an arbitrary horizontal deformation equal to 50 mm. The resulting Von Mises stresses are given in Figure 13, showing that no significant stresses result in this load case, which implies that the hinges in the frames are modeled well.

**Figure 14:**
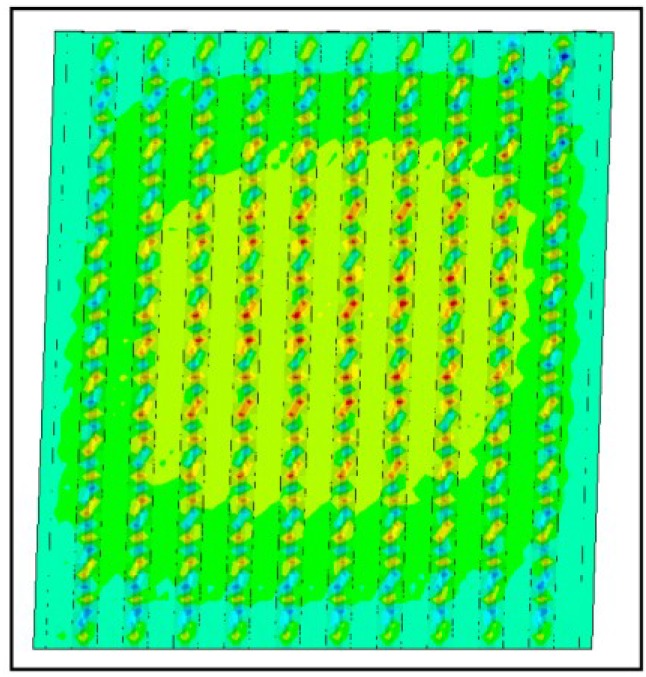
Global buckling.

**Figure 15:**
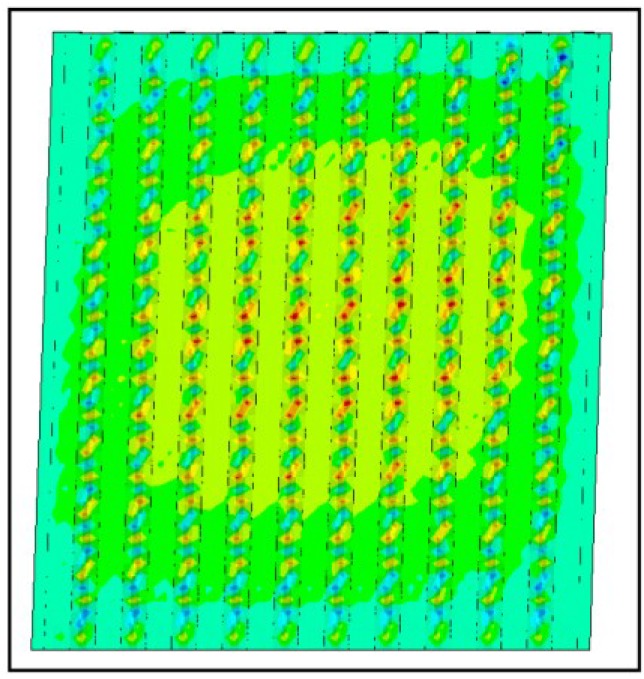
Local buckling.

**Figure 16:**
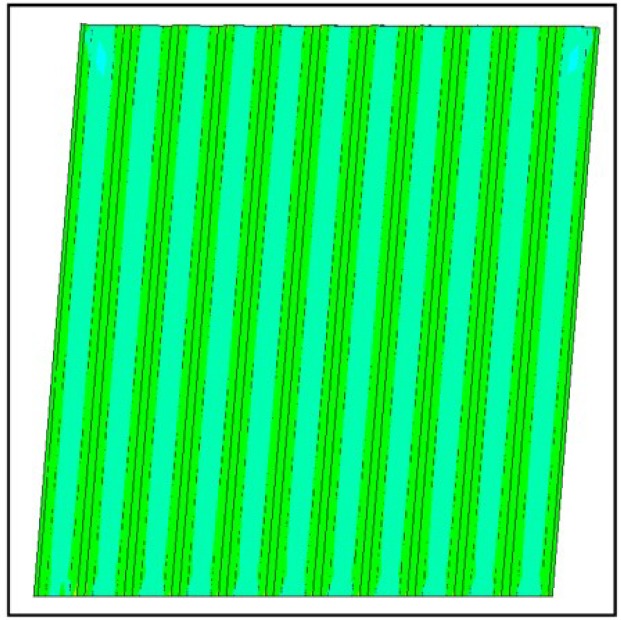
Heat affected zone (HAZ) yielding.

In [7] several failure modes are distinguished:

- global panel buckling, governed by buckling of the hat sections parts (Figure 14: out-of-plane deformations at the ultimate limit state;
- local panel buckling, governed by local buckling of the flat parts between the sections (Figure 15: out of plane deformations at the ultimate limit state);
- yielding of panel material in the HAZ zone (Figure 16, Von Mises stresses at the ultimate limit state).

As the optimized panel is supposed to be a Class 3 section, the second failure mode will not occur in practice for the considered profiles.

The FEM analysis is carried out in three steps:

- linear elastic analysis (LEA);
- linear local buckling analysis (LPA);
- geometrically and physically non-linear analysis (GMNIA: Geometrically and Materially Nonlinear Analysis with Imperfections).

LEA determines the best mesh dimensions needed for reliable results. LPA determines the magnitude and mode of the geometric imperfection model, which is generally based on the superposition of one or more local buckling modes. Finally, GMNIA results in solutions using geometrical as well as physical non-linearity.

**Figure 17:**
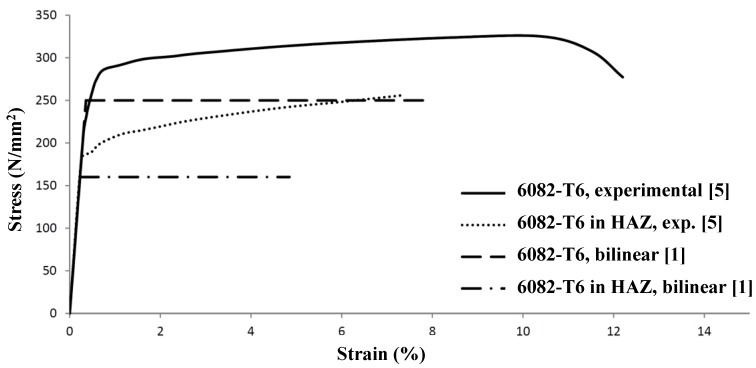
Stress-strain diagram of aluminum alloy 6082-T6.

In the FEM analysis, the material behavior of the 6082-T6 alloy is based on the experimentally determined stress-strain relationship of Scialpi [8]. A comparison between the bi-linear Eurocode 9 model without strain hardening [1] and the Scialpi model [8] is shown in Figure 17, where the width of the FSW heat-affected zone is supposed to be equal to the width of an MIG welded heat-affected zone, *i.e.*, 20 mm for a plate thicknesses up to 6 mm and 30 mm for a plate thicknesses between 6 and 12 mm.

**Figure 18:**
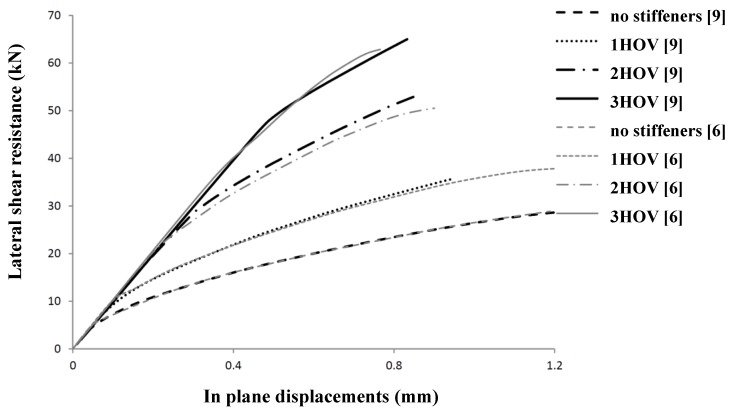
Comparison of lateral shear resistance for several geometries.

The numerical model is further verified by comparison to numerical research on the influence of stiffeners on steel shear panels [9,10]. Figure 16 shows the agreement between the Pater analysis [2] and the Alinia analysis [9,10], using the same geometrical and physical properties. As Figure 18 shows, the agreement is 100% when no stiffeners are used. The small difference for panels with stiffeners can be clarified by the use of SHELL elements in the Pater model *versus* BEAM elements in the Alinia model.

## 6. Parametric Studies

Parametric studies are carried out in order to analyze the influence of imperfections, edge beams and plate stiffeners on the resistance of the investigated shear panels.

**Figure 19:**
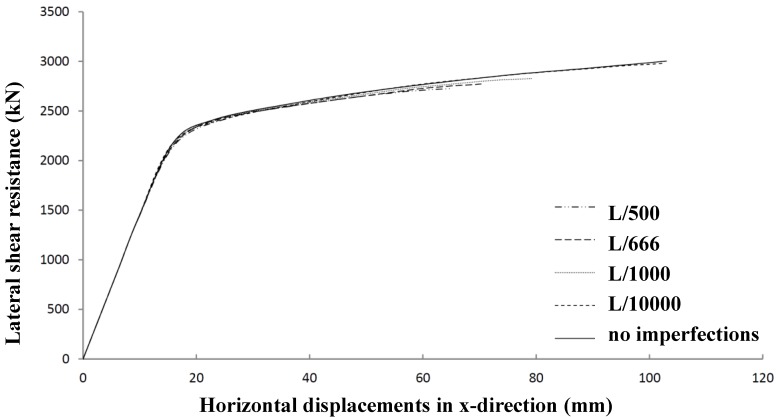
Influence of geometrical imperfections on lateral shear panel resistance.

The influence of the magnitude of geometrical imperfections is given in Figure 19, which shows that this influence is very small. Rather arbitrarily, an imperfection of 1/666 of the span length is chosen to be representative for further research.

**Figure 20:**
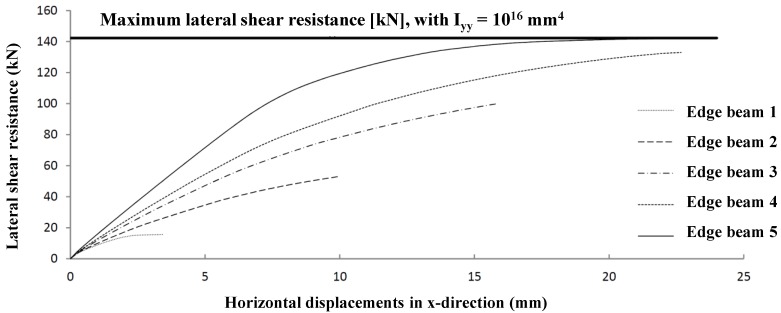
Lateral shear resistance for edge beams differing in second moment of inertia I_yy_: Edge Beam 1: I_yy_ = 1 mm^4^; Edge Beam 2: I_yy_ = 1 × 10^5^ mm^4^; Edge Beam 3: I_yy_ = 1 × 10^6^ mm^4^; Edge Beam 4: I_yy_ = 5 × 10^7^ mm^4^; Edge Beam 5: I_yy_ = 1 × 10^8^ mm^4^.

However, the influence of the stiffness properties of the edge beams is relatively high (see Figure 20). The influence has been investigated for five different edge beams, only differing in the second moment of inertia, I_yy_. Other properties (cross-section A and second moment of inertia I_zz_) are the same for the considered calculations. The maximum lateral resistance can only be reached by edge beams too stiff for practical situations.

For simplicity, the study on the influence of the edge beam stiffness was carried out on smaller plate dimensions, which of course resulted in lower values of the absolute lateral shear resistance, as well as horizontal displacements (compare with Figure 19).

**Figure 21:**
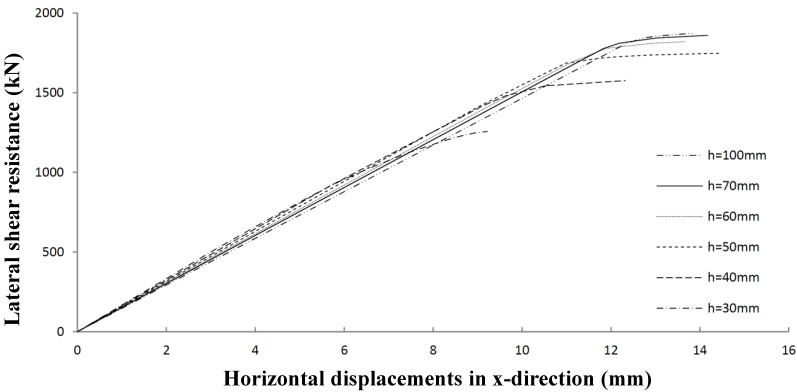
Lateral shear resistance of Panel 4 with varying profile heights.

At last, the influence of the height of the stiffeners using stiffener Models 2 and 4 (see Figure 8 and Figure 10) is investigated. Figure 21, which is worked out for Panel 4, shows that lateral shear resistance hardly increases when the height of the profiles is larger than 60 mm, which seems to be the upper limit for shear panel resistance.

**Figure 22:**
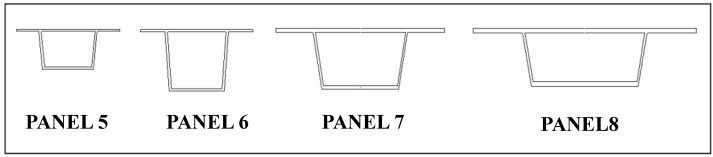
Geometry of optimized Shear Panels 5 to 8.

**Figure 23:**
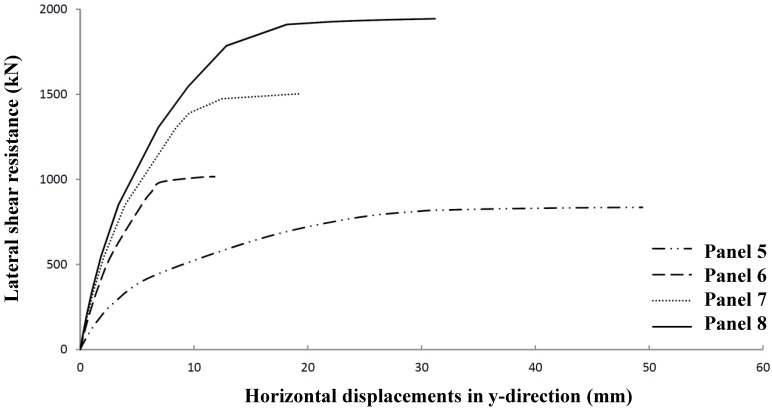
Lateral shear resistance *versus* in-plane deformations for Panels 5 to 8.

**Figure 24:**
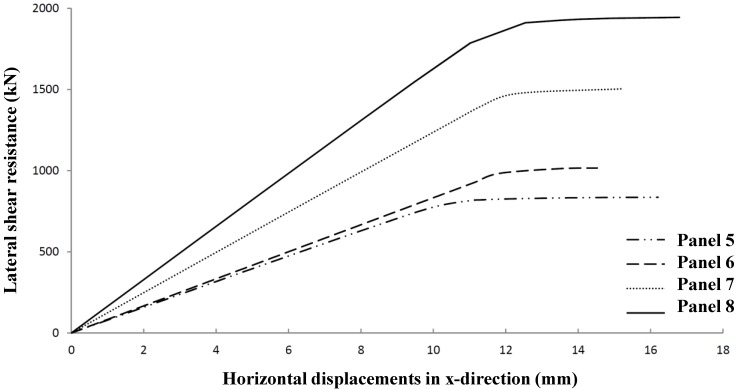
Lateral shear resistance *versus* out of plane deformations for Panels 5 to 8.

Determining optimum shear stiffened plates, Panel 4 has been further optimized to Panel Geometries 5 to 8 (Figure 22 and Table 10). The relevant shear resistances and its typical deformation behavior are given in Figure 23 and Figure 24.

**Table 10:** Dimensions of optimized Shear Panels 5 to 8.

Panel property	Panel 5	Panel 6	Panel 7	Panel 8
Length (mm)	4,000	4,000	4,000	4,000
Width (mm)	4,012	4,096	4,032	4,064
*a*_1_ (mm)	32	35	53	70
*a*_2_ (mm)	28	30	45	60
*a*_4_ (mm)	27	29	43	57
*t*_1_ (mm)	2.5	2.7	4.1	5.4
*t*_2_ (mm)	2.5	2.7	4.1	5.4
*t*_3_ (mm)	2.5	2.5	2.5	2.5
*h* (mm)	43	68	65	61
ρ_c;g_	0.35	0.64	0.64	0.64
ρ_o,HAZ_	0.64	0.64	0.64	0.64

## 7. Comparison between Design Rules and FEM Results

**Figure 25:**
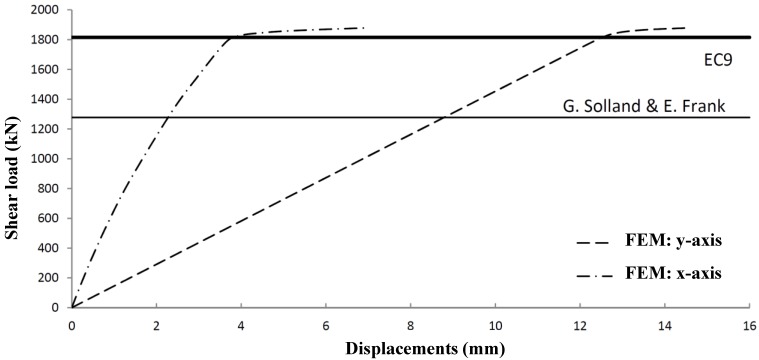
Load *versus* deformations Panel 4.

Figure 25 shows the shear panel resistance of Panel Type 4 using three different analysis methods: design rules according to Eurocode 9 [1], numerical analysis using ANSYS [2] and rational design according to [11]. Figure 25 also clearly shows that lateral shear resistance is governed by the plastic capacity of the panels. Global buckling instability is not governing for the considered panel types, while local buckling was already excluded by the application of a wall thickness not smaller than 2.5 mm. The advised rules according to Solland and Frank [11] are very safe.

**Figure 26:**
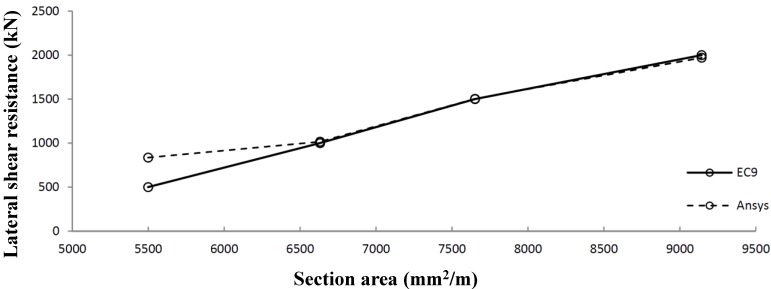
Comparison of shear panels optimized according to Eurocode 9 (EC9) *versus* ANSYS.

A comparison of Eurocode 9 *versus* ANSYS results shows very good agreement for Panel Types 6 to 8. Very small deviations occur due to geometrical imperfections used in the FEM model. Panel 5 shows a relatively large difference due to a deviating failure mode (global instability). The results have been worked out in a graph (Figure 26), which shows the lateral shear panel strength dependent on the cross-sectional panel.

## 8. Conclusions

The optimized cross-sectional design for shear panels applied in living areas on oil platforms has resulted in two section geometries: Panel 2 for wind load governing and Panel 4 (Figure 9 and Figure 11) for explosion and/or floor load governing. A comparison with existing shear panels leads to a material reduction of 10% to 25%. The optimization has been worked out for Class 3 cross-sections, using a minimum wall thickness of 2.5 mm.

Parametric studies show that the influence of geometric imperfections on the load bearing strength is very small. However, the stiffness of edge beams is significant. To reach the maximum lateral shear strength, the edge beam stiffness should be very high, resulting in unrealistic beam dimensions.

The failure mode depends on the height of the hat stiffeners. For sections using relatively high hat stiffeners, failure is introduced by the yielding of the heat-affected zone. For this type of cross-section, Eurocode 9 design rules and numerical calculations show very good agreement. For sections using relatively low hat stiffeners, failure is introduced by global buckling. For this type of cross-section, Eurocode 9 gives rather conservative results.

## 9. Recommendations

It is recommended to investigate the shear strength for panels with relatively low stiffener heights further by analytical and/or experimental research. For these panels, global buckling of the stressed skin panels determines the ultimate limit strength. The Eurocode 9 design rules seem to be rather conservative for this type of panel. Further it is recommended to expand the research to Class 4 cross-sections, which will reduce the optimized cross-sectional area even more.

## References

[1] NEN-EN 1999 Part 1.1, Eurocode 9 Design of aluminium structures General rules and rules for buildings, 2007..

[2] Pater G. (2011). Optimization of aluminium stressed skin panels in off shore applications. M.Sc. Thesis.

[3] Virag Z. (2004). Optimum design of stiffened plates for different loads and shapes of ribs. J. Comput. Appl. Mech..

[4] Ogle M.H., Maddox S.J., Threadgill P.L. (1998). Joints in Aluminium. Proceedings of the 7th International Aluminium Conference.

[5] (1999). NORSOK N-003. Nordic standard for actions and action effects.

[6] Soetens F., van Hove B.W.E.M. (2013). Optimization of Aluminium Stressed Skin Panels in Offshore Applications. Proceedings of the 12th International Aluminium Conference.

[7] Sun H.H., Spencer J. (2005). Buckling strength assessment of corrugated panels in offshore Structures. Mar. Struct..

[8] Scialpi A., de Filippis L.A.C., Cavaliere P. (2007). Influence of shoulder geometry in microstructure and mechanical properties of friction stir welded 6082 aluminium alloy. Mater. Des..

[9] Alinia M.M., Shirazi R. (2009). On the design of stiffeners in steel plate shear walls. J. Constr. Steel Res..

[10] Alinia M.M. (2005). A study into optimization of stiffeners in plates subjected to shear loading. Thin Walled Struct..

[11] Solland G., Frank E. Rational Design of Stressed Skin Offshore Modules. Proceedings of the 5th International Conference on Behavior of Offshore Structures.

